# Maternal Antibody Interference and Vaccination Gaps in Goat Kids: Implications for Peste des Petits Ruminants Eradication

**DOI:** 10.3390/vaccines14100855

**Published:** 2026-09-28

**Authors:** Abdul Kabir, Amjad Hussian Mirani, Reham Karam, Ahmed K. Farag, Tinatin Elbakidze, Ahmed M. A. Meligy, Saad Shousha, Yassir A. Almofti, Muhammad Shahzad Chohan, Moustafa Sarhan, Mahmoud G. El Sebaei

**Affiliations:** 1 Department of Veterinary Microbiology, Faculty of Animal Husbandry and Veterinary Sciences, Sindh Agriculture University, Tandojam 70060, Pakistan; 2 Department of Veterinary Medicine, Faculty of Animal Husbandry and Veterinary Sciences, Sindh Agriculture University, Tandojam 70060, Pakistan; miramjadsau@gmail.com; 3 Virology Department, Weqaa Center Central Laboratory, Riyadh 14714, Saudi Arabia; reham_farag@mans.edu.eg; 4 Virology Department, Faculty of Veterinary Medicine, Mansoura University, Mansoura 35516, Egypt; 5 Sygnature Discovery, 2350 rue Cohen Suite 201, Montréal, QC H4R 2N6, Canada; akm190@gmail.com; 6 One Health Institute, School of Science and Technology, University of Georgia, Tbilisi 0171, Georgia; t.elbakidze@yahoo.com; 7 Central Lab., Department of Clinical Sciences, College of Veterinary Medicine, King Faisal University, Al-Ahsa 31982, Saudi Arabia; amelegi@kfu.edu.sa; 8 Department of Biomedical Sciences, College of Veterinary Medicine, King Faisal University, Al-Ahsa 31982, Saudi Arabia; smmohamed@kfu.edu.sa (S.S.); yamofti@kfu.edu.sa (Y.A.A.); 9 Department of Biomedical Sciences, College of Clinical Pharmacy, King Faisal University, Al-Ahsa 31982, Saudi Arabia; mshwhan@kfu.edu.sa (M.S.C.); mmsarhan@kfu.edu.sa (M.S.)

**Keywords:** Peste des petits ruminants, maternal antibodies, colostrum, vaccination interference, goat kids, serosurveillance, eradication, herd immunity, PPR vaccination

## Abstract

Peste des Petits Ruminants (PPR) is an economically devastating transboundary viral disease of small ruminants across Africa, Asia, and the Middle East. Despite effective live attenuated vaccines and the FAO/WOAH global eradication campaign targeting 2030, recurrent outbreaks persist, particularly among young goats. A major underrecognized obstacle is maternal antibody (MA) interference in goat kids, which compromises early-life vaccination and creates hidden immunity gaps within vaccinated herds. Maternal antibodies, acquired exclusively via colostrum, provide temporary passive protection but can neutralize vaccine virus and reduce effective seroconversion. This creates a critical “window of susceptibility” (typically 2–5 months of age) during which goat kids are neither protected against field infection nor responsive to vaccination. Reported estimates vary from approximately 70–100 days to detectability for up to 6 months, depending on the endpoint, assay, and study population. Conventional serosurveillance overestimates herd immunity because seropositive goat kids with residual MAs are falsely classified as protected. Fixed-age mass vaccination campaigns thus fail to achieve effective immunity in the most vulnerable age group. Strategies to overcome MA interference include delayed primary vaccination, booster schedules, targeted immunization, intranasal vaccination, and recombinant vaccines. Major barriers include poor farmer awareness, inadequate cold-chain infrastructure, and transboundary animal movement. Rapid farm turnover represents a separate operational barrier that further complicates vaccination timing. Achieving PPR eradication by 2030 requires a paradigm shift toward age-specific immunity in goat kids. Age-stratified protocols, farmer education, standardized diagnostic thresholds, rapid pen-side MA tests, and longitudinal studies in underrepresented regions including Pakistan are urgently needed.

## 1. Introduction

The Peste des petits ruminants (PPR) is a highly contagious, fatal Morbillivirus disease affecting sheep, goats, and other small ruminants [1]. It is endemic across Africa, Asia, and the Middle East, causing annual economic losses estimated at US $1.4–2.1 billion [2]. The disease disproportionately impacts poor and marginal farmers who depend on small ruminants for food and income [3]. In 2015, the Food and Agriculture Organization (FAO) and the World Organisation for Animal Health (WOAH) launched a global strategy to eradicate PPR by 2030, modeled after the successful rinderpest eradication campaign. Vaccination with live attenuated vaccines (primarily the Nigeria 75/1 strain) is the cornerstone of this strategy [4]. However, a critical barrier to eradication is the interference of maternal antibodies (MAs) with early-life vaccination in goat kids [5]. MAs acquired via colostrum provide passive protection but also neutralize live vaccines, creating an immunity gap. This review synthesizes current knowledge on the duration and determinants of maternal antibodies against PPR in goat kids, identifies vaccination gaps, and proposes context-specific strategies to overcome this barrier. Despite the availability of an effective vaccine, persistent transmission in endemic regions highlights critical gaps in early-life immunity, particularly in goat kids.

## 2. Window of Susceptibility in Goat Kids

The window of susceptibility is a variable period during which maternal antibodies (MAs) have declined below protective levels but remain high enough to neutralize live attenuated vaccines. During this interval, goat kids are vulnerable to PPR infection even if vaccinated [6]. 

MAs decay exponentially in goat kids, with the rate of decline varying depending on initial titers and individual factors [7]. The susceptibility window generally opens between 2 and 5 months of age, depending on initial MA titers and decay rates [8]. However, the timing is highly heterogeneous and is influenced by multiple factors, including colostrum transfer efficiency, dam vaccination status and timing, breed, management practices, exposure pressure, vaccine characteristics, and the protective threshold used to define susceptibility.

Reported timing varies considerably across studies. Ref. [7] reported the window opening at 28 days, with all goat kids seronegative by 100 days [9] observed onset at 105 days and complete susceptibility at 150 days. Ref. [10] found onset at 120 days and full susceptibility at 150 days. These discrepancies reflect differences in vaccine strains, dam immunity, colostrum management, breed, and environmental conditions. Conceptually, the window exists between two thresholds on the declining MA curve: the protective threshold (below which infection risk begins) and the interference threshold (below which vaccination becomes effective). The gap between these thresholds is the susceptibility window. These variations highlight that the window is not a fixed biological constant but a highly context-dependent period. These variations highlight that the window is not a fixed biological constant but a highly context-dependent period. The duration and timing of the susceptibility period vary with multiple interacting factors, including maternal antibody levels and decay rates (influenced by colostrum quality and dam vaccination history), host factors such as breed, age of dam, nutrition, and sex, vaccine characteristics including strain, dose, route, and frequency, management and environmental factors such as colostrum feeding practices, exposure pressure, and endemicity, and the diagnostic criteria used to define protective antibody levels.

It is important to distinguish three different serological concepts. The protective threshold refers to an antibody level associated with protection against clinical disease, preferably supported by neutralizing antibody measurements and challenge or field evidence. The seronegativity threshold refers to the assay-specific level below which a binding assay such as c-ELISA classifies an animal as seronegative. Seronegativity therefore indicates loss of detectable antibody reactivity in that assay but does not necessarily indicate complete loss of neutralizing protection. The interference threshold, in contrast, refers to the antibody level above which residual maternal antibodies prevent or substantially impair the induction of active immunity following administration of live attenuated PPR vaccine.

Critically, the interference threshold has not been experimentally defined for PPR in goat kids. No identified study directly establishes the antibody titre above which live PPR vaccination fails to induce an active immune response in kids. Consequently, the time to c-ELISA seronegativity should not be interpreted as either the protective threshold or the interference threshold. Establishing this relationship is one of the most consequential unresolved questions for evidence-based vaccination scheduling.

Figure 1 provides a conceptual illustration of this dynamic; the transitions shown are approximate and should not be interpreted as fixed age cut-offs or specific vaccination recommendations.

### 2.1. Literature Search Strategy

This narrative review was conducted following the Scale for the Assessment of Narrative Review Articles (SANRA) guidelines. A narrative approach was chosen to synthesize the heterogeneous body of literature on maternal antibody interference in goat kids across epidemiological, immunological, and operational domains. A comprehensive literature search was performed in PubMed, Google Scholar, Scopus, and Web of Science for articles published from January 1990 to July 2026, using the Boolean search strategy: (“Peste des petits ruminants” OR “PPR” OR “PPRV”) AND (“maternal antibodies” OR “maternal immunity” OR “colostrum”) AND (“goats” OR “kids” OR “small ruminants”) AND (“vaccination” OR “vaccine interference”). Inclusion criteria comprised peer-reviewed original research, review articles, and official FAO/WOAH reports reporting on maternal antibody dynamics or PPR vaccination in goats or sheep, published in English. Conference abstracts, theses, non-English articles, and duplicate publications were excluded. Titles and abstracts were screened independently by two reviewers; full texts of potentially eligible articles were assessed against inclusion criteria, with disagreements resolved through consensus. Reference lists of included studies were manually searched for additional citations. Key data were extracted narratively; no meta-analysis or quality scoring was performed due to study heterogeneity and the narrative design of this review.

### 2.2. Maternal Antibodies in Goat Kids

Because the caprine placenta is epitheliochorial, negligible immunoglobulin is transferred in utero. Therefore, newborn goat kids acquire maternal antibodies (MAs) exclusively via colostrum intake within the first 24–48 h of life, when gut closure occurs [13]. Colostral antibodies reflect the dam’s immune status, making pre-partum vaccination of does critical for passive protection of offspring. MAs provide essential protection against PPR during the first weeks of life, when the kid’s own immune system is immature. However, MAs also interfere with live attenuated PPR vaccines by neutralizing the vaccine virus before it can replicate and induce active immunity [14]. This interference creates a window of susceptibility—a period when MAs are too low to protect against field infection but too high to permit effective vaccination.

In the studies reviewed, goats appeared to exhibit lower and shorter-lasting MAs against PPR compared to sheep [15]. This difference may be due to species-specific differences in colostral antibody transfer efficiency and antibody catabolism rates, although direct comparative studies are limited and the findings should be interpreted with caution. This makes goat kids particularly vulnerable to vaccination gaps and underscores the need for species-specific vaccination schedules.

### 2.3. Immunological Mechanisms of Maternal Antibody Interference

Maternal antibodies interfere with vaccination through multiple immunological mechanisms. Understanding these mechanisms is essential for developing strategies to overcome MA interference.

#### 2.3.1. FcRn and Colostral IgG Transfer

In ruminants, colostral IgG is predominantly IgG1, which is selectively transported into mammary secretion. Neonatal Fc receptor (FcRn) mediates IgG transcytosis across neonatal enterocytes before gut closure, facilitating the absorption of colostral IgG into the kid’s circulation. FcRn also protects IgG from intracellular degradation, extending its half-life in circulation. The catabolic half-life of caprine IgG is determined by FcRn-mediated recycling; in goats, IgG half-life is approximately 14 days, comparable to other ruminants. This decay constant directly determines the rate of maternal antibody decline in goat kids. The exponential decay of maternal antibodies can be modeled as:

Ab(t) = Ab_0_·e^(−kt)^

where Ab(t) is the antibody level at time t, Ab_0_ is the initial antibody level at birth (influenced by colostrum intake), and k is the decay constant (determined by IgG catabolic half-life). This framework explains why kids with higher initial MA titers (from adequate colostrum) have a longer period of passive protection and potentially prolonged interference with vaccination.

#### 2.3.2. Neutralization of the Live Vaccine Virus

Maternal antibodies, primarily of the IgG isotype, bind to the live attenuated PPR vaccine virus with high affinity. This binding prevents the virus from attaching to and entering host cells, thereby blocking viral replication. Without replication, the vaccine virus cannot produce sufficient antigen to stimulate the immune system, resulting in poor or absent seroconversion [16]. Neutralization of vaccine virus by pre-existing maternal antibodies is supported by experimental evidence from PPR vaccination studies and is considered the most directly supported mechanism of maternal antibody interference. The neutralizing capacity of MAs is directly proportional to their concentration in the kid’s circulation; higher MA titers result in more complete neutralization of the vaccine virus.

#### 2.3.3. Epitope Masking

PPRV neutralizing epitopes reside on the H (SLAM/CD150- and nectin-4-binding) and F glycoproteins. Epitope masking occurs when maternal antibodies bind to these critical epitopes, physically covering them. This occlusion of the H receptor-binding site blocks SLAM-mediated entry into lymphocytes, which is the primary site of vaccine virus replication. Epitope masking prevents B cells from recognizing the antigen and initiating an adaptive immune response. Even if the virus is not completely neutralized, the presence of bound antibodies can shield key antigenic sites from immune recognition, leading to reduced antibody production after vaccination [17]. This mechanism has been primarily documented in other viral systems (e.g., measles, influenza) and represents an immunologically plausible mechanism that may also contribute to MA interference in PPR. However, direct PPR-specific evidence remains limited and warrants further investigation. This mechanism is particularly problematic for live attenuated vaccines, as the immune system may fail to recognize the vaccine virus as a foreign antigen.

#### 2.3.4. Fc Receptor-Mediated Immune Suppression

Antigen–antibody complexes formed between the vaccine virus and maternal antibodies can interact with inhibitory Fc receptors, particularly FcγRIIB, on B cells. This inhibition requires co-ligation of the B cell receptor (BCR) and FcγRIIB by IgG-containing immune complexes. Co-ligation leads to phosphorylation of the immunoreceptor tyrosine-based inhibition motif (ITIM) and recruitment of SHIP-1 (SH2-containing inositol phosphatase-1), which attenuates BCR signaling, thereby suppressing B-cell activation, proliferation, and antibody production. This interaction delivers a negative signal that suppresses B-cell activation, proliferation, and antibody production. This is a key mechanism through which maternal antibodies actively suppress the immune response to vaccination, rather than simply neutralizing the vaccine virus [16]. This mechanism, well characterized in other viral systems, suggests that maternal antibodies may actively suppress immune responses rather than merely neutralizing the vaccine virus. While this is an immunologically plausible mechanism in PPR, direct evidence in goat kids following PPR vaccination is currently lacking and requires experimental confirmation. This suppression may persist even after antibodies have declined to sub neutralizing levels, though this observation is extrapolated from studies in other viral systems and requires confirmation in the context of PPR [16].

#### 2.3.5. Effects on Cellular Immunity

Maternal antibodies also affect cellular immune responses. Neutralization of the vaccine virus reduces antigen presentation by antigen-presenting cells, leading to weaker activation of CD4^+^ helper T cells and CD8^+^ cytotoxic T cells [18]. Humoral responses are consistently suppressed by maternal antibodies. However, evidence from related morbilliviruses (e.g., measles and canine distemper) suggests that T-cell priming may occur even when antibody responses are abolished. The extent of cellular impairment in PPRV-vaccinated kids is unresolved. These effects on cellular immunity have been observed in other viral systems, but their relevance to PPR vaccination in goat kids remains hypothetical. Further PPR-specific studies are needed to determine the extent to which maternal antibodies impair cellular immune responses. Consequently, both humoral and cellular arms of the immune system are compromised, resulting in reduced immunological memory and shorter duration of protection [19].

#### 2.3.6. Why Live Attenuated PPR Vaccines Fail in the Presence of Maternal Antibodies

Collectively, these mechanisms explain why live attenuated PPR vaccines fail in the presence of maternal antibodies. The vaccine virus is may be neutralized, masked, or actively suppressed, preventing it from inducing a protective immune response [20]. The FcRn-mediated decay framework (Ab(t) = Ab_0_·e^(−kt)^) provides a quantitative basis for understanding this process: higher initial MA titers require longer decay periods before the interference threshold is crossed, delaying the optimal vaccination age. These mechanisms may contribute to reduced seroconversion and potentially increase the period during which goat kids remain susceptible to infection. This leads to poor seroconversion, shorter duration of immunity, and a window of susceptibility during which goat kids are vulnerable to field infection despite being vaccinated The severity of interference depends on MA titers, which vary with colostrum quality, dam vaccination status, breed, and management. Importantly, while the mechanistic framework is well supported by evidence from other viral systems, PPR-specific studies are needed to confirm the contributions of epitope masking, Fc receptor-mediated suppression, and cellular immunity interference in goat kids.

## 3. Determinants of Maternal Antibody Persistence

The level and duration of maternal antibodies (MAs) against PPR in goat kids are variable across reviewed studies; study-specific estimates are reported consistently throughout the manuscript. This variability is determined by four categories of factors: vaccine-related, host-related, viral and environmental, and diagnostic. Maternal antibodies against the virus can be detected in young animals and remain able to neutralize the virus for three to four months enabling a level of protection in newborn animals [21]. Therefore, the timing of vaccination should consider the persistence and functional activity of maternally derived antibodies rather than relying solely on a fixed age [22].

Another issue in PPR vaccination is the time of vaccination of young animals. There are few reports on duration of persistence of maternal antibodies in lambs/kids born to vaccinated dams. Maternal antibodies in young animals have been reported to remain detectable for up to 6 months of age but fell below the protection threshold level at 3.5 months in lambs and 4.5 months in goat kids [15].

Similarly, maternal antibodies in kids vaccinated with a lineage IV vaccine declined from the third month onward and were reported to fall below the protective level by approximately the fourth month [6].

Based on these findings, vaccination at approximately 4 months has been proposed in some earlier studies to reduce the risk of a prolonged susceptibility period [6]. However, this timing should not be considered a universal recommendation because maternal antibody kinetics and vaccine responsiveness vary among animals and production systems. The optimal vaccination age should therefore be determined according to maternal antibody persistence, breed, colostrum management, local epidemiological conditions, and transmission risk [7]. A candidate strategy involving delayed primary vaccination at 5–6 months followed by a booster at 8–9 months may help address maternal antibody interference; however, this approach requires field validation before it can be considered for broader adoption [23].

### 3.1. Vaccine-Related Factors

Vaccine-related determinants of MA duration are summarized in Table 1. Vaccine strain influences MA persistence. The Nigeria 75/1 strain induces higher and longer-lasting MAs (up to 7 months) compared to Sungri/96 (up to 5 months) [21]. The Egypt/87 strain has been shown to induce MA persistence for up to 4 months in kids [12]. Egypt/87 is a locally developed Egyptian vaccine strain, distinct from the Indian Arasur/87 strain.

Vaccine dose affects MA titers. Higher doses (e.g., 1000 TCID50) produce stronger MAs but also prolong interference, delaying the window for effective kid vaccination [6].

Route of administration matters. Studies in adult goats have compared subcutaneous and intranasal routes, with subcutaneous administration generally inducing higher antibody responses [24]. However, direct evidence comparing intramuscular and subcutaneous routes in does and their kids remains limited.

**Table 1:** Vaccine-related determinants of maternal antibody duration.

Study	Vaccine Strain	Route	Vaccination Frequency (Dam)	Reported MA Duration in Goat Kids	Key Findings
[23]	Sungri/96	1 mL/SC	Single	Up to 5 months	Higher pre-vaccination maternal antibody titre negatively affects 28-day post-vaccination response; 94.9% goat kids protected post-vaccination
[7]	Nigeria 75/1	1 mL/SC	Single	70–100 days	First kid seronegative at 28 days; all seronegative by 100 days
[25]	BLRI PPR Vaccine	1 mL/SC	Single	Up to 4 months	goat Kids maintained protective level (51% PI) up to 4 months; vaccinating at 4 months recommended
[15]	Nigeria 75/1	Not specified	Single	Lambs: 3.5 months; Kids: 4.5 months	Detectable up to 6 months; fell below protection threshold at 3.5–4.5 months in goat kids
[6]	Asian Lineage IV	Not specified	Single	4–5 months	Passive immunity wanes after 4–5 months; maternal antibodies affect vaccine response

Frequency of vaccination in does boosts MA levels. A single dose of the Nigeria 75/1 vaccine induces protective immunity in adult animals for up to 36 months, whereas in young animals (1.5 and 3 months of age), the immune response persists for a shorter duration—approximately 4 to 6 months indicating the importance of age-specific vaccination strategies [23]. Furthermore, multiple vaccination schedules (e.g., three doses at 3-month intervals) have been reported to influence the duration of MA persistence, although the reported durations vary across studies [23]. A key issue is timing mismatch: when goat kids are vaccinated according to a fixed schedule (e.g., at 4 months), those with persistently high MAs fail to seroconvert, while those with waning MAs become susceptible to field infection before the vaccine takes effect This highlights the need for individualized vaccination timing based on MA kinetics and local epidemiological conditions.

### 3.2. Host-Related Factors

Host-related factors affecting MA persistence are summarized in Table 2. Colostrum intake quality is the single most important host factor. goat Kids that receive insufficient colostrum or delayed colostrum intake have lower initial MA titers and shorter protection. Conversely, adequate colostrum from well-vaccinated does provides high MAs but prolongs interference.

Nutrition status of both dam and kid affects MA levels. Malnourished dams produce lower-quality colostrum with reduced immunoglobulin concentrations. Malnourished kids exhibit faster MA decay due to compromised metabolic and immune function.

Maternal antibody persistence varies with breed and sex, as documented in studies on small ruminants. Limited evidence suggests that female kids may maintain MAs slightly longer than males, but this finding requires further confirmation. Certain breeds exhibit longer-lasting MAs compared to others. The timing of dam vaccination relative to parturition influences colostral antibody concentration, with vaccination 4–6 weeks’ pre-partum being optimal for passive transfer [6]. In the studies reviewed, goats appeared to exhibit lower and shorter-lasting MAs against PPR compared to sheep [21]. This difference may be due to species-specific differences in colostral antibody transfer efficiency and antibody catabolism rates, although direct comparative studies are limited and the findings should be interpreted with caution.

### 3.3. Viral and Environmental Factors

Circulating PPRV strains affect the interaction between maternal antibodies and field challenge. Maternal antibody persistence varies with circulating virus lineages; in goats vaccinated with lineage IV vaccines, antibodies remain detectable up to 6 months but decline below protective levels by 4 months of age [6]. goat Kids with sub protective MA titers may resist infection from low-virulence strains but succumb to highly virulent lineages.

Endemic settings influence exposure risk. In areas with frequent field challenge, natural boosting of maternal antibodies in does may occur; however, this also increases infection risk for goat kids during the susceptibility window.

Management systems significantly impact vaccination success. Intensive systems allow controlled, uniform vaccination schedules with accurate age recording. In contrast, pastoral and extensive systems face substantial challenges, including mixed-age herds, unknown vaccination histories, poor colostrum management, and difficulty implementing age-based vaccination protocols [23].

### 3.4. Diagnostic Tools for Assessing Maternal Immunity

Diagnostic tools for assessing maternal immunity are compared in Table 3. Competitive ELISA (c-ELISA) is the most common field test for PPR antibodies. It is rapid, cost-effective, and suitable for large-scale surveillance. In a Studiey evaluating diagnostic performance in goats, c-ELISA demonstrated (100%) sensitivity and (88.24%) specificity compared to VNT, with almost perfect agreement (κ = 0.83) [28]. However, diagnostic accuracy depends on multiple factors, including the specific assay used, the antigen preparation, the reference standard, the population tested, and the cutoff applied. Therefore, these performance metrics should not be generalized across all c-ELISA formats without verification.

Crucially, c-ELISA cannot distinguish MAs from vaccine-induced antibodies, as c-ELISA detects antibodies that bind to or compete with the assay antigen or monoclonal antibody and provides a serological measure of antibody reactivity [6]. Neither c-ELISA nor VNT reliably distinguishes maternal from vaccine-derived antibodies. This is a major limitation when using these tests for vaccination timing, as seropositive goat kids with persistent maternal antibodies may be falsely classified as protected when they are actually susceptible once maternal antibodies decline.

Virus neutralization test (VNT) is the gold standard for measuring functional antibodies but requires cell culture and advanced laboratory facilities, making it impractical for field use [29]. The HPPR b-ELISA showed almost perfect agreement with VNT in goats (κ = 0.83), whereas the commercial ID Screen^®^ PPR c-ELISA showed substantial agreement with VNT (κ = 0.69) [28]. VNT remains the reference method for detecting neutralizing antibodies.

VNT measures functional neutralizing antibodies and is widely used as a reference assay for assessing neutralizing antibody responses. VNT detects neutralizing antibodies, whereas c-ELISA detects total antibodies (both binding and neutralizing).

Therefore, c-ELISA seronegativity should not automatically be interpreted as loss of neutralizing protection, and neither c-ELISA seronegativity nor loss of neutralizing antibodies alone establishes the antibody level at which maternal antibodies interfere with live vaccine take. These represent distinct immunological and operational endpoints that may occur at different times.

Cut-off challenges complicate cross-study comparisons. Protective thresholds vary (e.g., SN titre ≥ 1:8 by VNT; VNT titre > 1:8) with no universally accepted standard [15,30]. The choice of cutoff significantly affects the interpretation of serological results and must be considered when comparing studies.

Field applicability remains limited. Molecular pen-side tests exist for virus detection [31], but rapid serological tests for MA status are unavailable. Farmers cannot measure MA levels before vaccination, forcing reliance on fixed-age schedules that fail for some goat kids.

#### 3.4.1. Serological Approaches to Distinguish Maternal from Vaccine-Induced Antibodies

Several serological approaches used in other viral systems provide promising strategies for distinguishing passive from active antibody responses; however, their ability to discriminate maternal from vaccine-induced PPRV antibodies in goat kids has not yet been sufficiently validated.

#### 3.4.2. IgM-Specific ELISA

Systemically transferred maternal antibodies in ruminant kids are predominantly IgG, particularly IgG1. Therefore, the presence of IgM antibodies in a kid may indicate an active primary immune response rather than passively acquired maternal antibodies. IgM-specific ELISA is a promising approach for PPR serology, but it requires PPR-specific validation in goat kids before it can be recommended for routine use.

#### 3.4.3. IgG Avidity Index

Maternal IgG is affinity-matured and high-avidity due to previous antigen exposure. In contrast, a kid’s primary immune response yields low-avidity IgG. Avidity indices, measured using urea or thiocyanate elution, can distinguish high-avidity maternal from low-avidity vaccine-induced antibodies. This approach is established in measles and rubella serology; however, its application to PPR in goat kids remains to be validated.

#### 3.4.4. Paired Sera

Collecting paired sera 21–28 days apart allows assessment of longitudinal antibody kinetics using existing c-ELISA. A falling titre is consistent with decaying maternal antibodies, while a rising titre is consistent with vaccine take; however, infection can also produce an antibody rise, and paired sera alone cannot definitively distinguish maternal from vaccine-induced antibodies. This approach is field-deployable with existing c-ELISA infrastructure, although interpretation requires epidemiological context.

#### 3.4.5. Isotype/Subclass Profiling

Maternal antibodies in ruminants are predominantly IgG1, while active immune responses may involve other isotypes or subclasses. Profiling antibody isotypes or subclasses could provide additional discrimination between passive and active immunity, although this approach requires further validation for PPR.

#### 3.4.6. DIVA-Capable Vaccine Platforms

Differentiating infected from vaccinated animals (DIVA) vaccine platforms incorporate marker antigens to distinguish vaccine-induced antibodies from those induced by field infection. DIVA primarily addresses the infected-versus-vaccinated problem rather than the maternal-versus-vaccine problem and therefore represents a future complementary approach rather than a direct solution to maternal antibody interference.

These approaches convert the diagnostic problem from an unsolved issue into a concrete research and product-development agenda. The IgM and avidity approaches are particularly promising but require PPR-specific validation, while paired sera testing can be implemented immediately with existing c-ELISA infrastructure, provided results are interpreted with appropriate epidemiological context.

### 3.5. Colostrum Management as a Critical Modifiable Factor

Colostrum management is arguably the most practical and modifiable factor affecting maternal antibody levels in goat kids. The quantity, quality, and timing of colostrum intake directly determine initial MA titers, which in turn influence both protection duration and interference persistence. However, while colostrum management is highly practical at the individual farm level, its implementation in large-scale vaccination programs faces logistical constraints and is not without risks to neonates, as summarized in Table 4. Optimal colostrum feeding protocols recommend 10–15% of body weight (approximately 300–450 mL for a 3 kg kid) within the first 24 h of life, with the first feeding within 1–2 h of birth, as antibody absorption declines rapidly after 6 h [32]. Colostrum from dams vaccinated against PPR within 2–6 months’ pre-partum contains the highest antibody concentrations [26].

Quality assessment of colostrum can be performed using a colostrometer or Brix refractometer. Colostrum with >50 mg/mL IgG (or >22% Brix) is considered high quality [27]. Farmers should be trained to assess colostrum quality and prioritize feeding high-quality colostrum to all goat kids. Practical recommendations include vaccinating does 4–6 weeks before expected parturition, ensuring goat kids suckle within 1–2 h of birth, feeding colostrum from the dam or a vaccinated donor, avoiding pooling of colostrum from multiple dams (which increases disease transmission risk), and freezing excess high-quality colostrum for emergencies [27].

Optimal colostrum management ensures consistent MA levels across goat kids, reducing variability and allowing more predictable vaccination schedules. However, it does not eliminate interference; it simply makes MA dynamics more predictable and manageable.

### 3.6. Evidence Hierarchy for Factors Influencing Maternal Antibody Persistence

The evidence hierarchy for factors influencing maternal antibody persistence is presented in Table 5. To critically integrate the extensive list of factors identified in this review, we have categorized them according to the strength and directness of the available evidence. This hierarchy is based on the availability of PPR-specific studies, study design (experimental vs. observational), sample size, and consistency of findings across studies. The classification is as follows: Strong Evidence (supported by multiple studies with consistent findings), Moderate Evidence (supported by some studies but with limitations), Weak/Limited Evidence (supported by few studies or single reports), and Speculative/Hypothetical (plausible but lacking direct PPR-specific evidence).

## 4. Implications for Herd Immunity and PPR Eradication

Maternal antibody interference directly undermines herd immunity—the proportion of protected individuals required to interrupt transmission. The PPR eradication strategy commonly targets at least 70% post-vaccination immunity in endemic small-ruminant populations [33]. However, this target should not be interpreted as a universal biological threshold because the proportion of protected animals required to interrupt transmission depends on the transmission potential of the virus and the structure and heterogeneity of the host population.

The theoretical herd immunity threshold can be derived from the basic reproduction number (R_0_) as:

pc = 1 − 1/R_0_

Under homogeneous mixing and uniform vaccine-derived protection, an R_0_ of 2.0 corresponds to a critical vaccination proportion of 50%, an R_0_ of 3.0 to 67%, and an R_0_ of 3.5 to 71%. Thus, thresholds approaching 70% are compatible with populations in which the effective R_0_ is approximately 3.3 or higher. However, these calculations represent theoretical values under simplified assumptions and should not be interpreted as a fixed PPRV threshold across epidemiological settings.

Vaccination failure in endemic regions occurs when goat kids are vaccinated according to national campaigns (e.g., at 3–4 months) but retain interfering MAs. These goat kids seroconvert poorly, remain unprotected, and contribute to the susceptible pool. Consequently, even high coverage rates (e.g., 90%) can translate into effective immunity far below the threshold [34].

This creates a hidden immunity gap (Figure 2). Serosurveys that detect antibodies cannot distinguish MAs from vaccine-induced immunity. A kid with persistent MAs tests positive but remains susceptible once MAs decline below protective levels. Such goat kids are falsely counted as protected, masking true herd immunity deficits [22].

Threshold immunity concept requires reconsideration. The static 70% threshold assumes uniform protection across age groups. In reality, goat kids under 6 months form a distinct subpopulation with unpredictable MA status. Vaccination programs that ignore this dynamic will consistently fail to reach effective herd immunity in kid cohorts [7].

Age and population heterogeneity can further modify the relationship between vaccination coverage and transmission interruption. Ref. [8] investigated age-specific force of infection among sheep, goats, and cattle in northern Tanzania and identified age-related patterns in PPRV exposure, although the overall evidence for strong age-specific heterogeneity was limited. Their findings therefore support consideration of age structure in PPRV transmission studies but do not justify assuming that a single age cohort universally sustains transmission. Ref. [35] further demonstrated that heterogeneity in small-ruminant populations can substantially influence vaccination effectiveness. Their modelling showed that the 70% post-vaccination immunity target may not reliably achieve elimination when vaccination is biased toward accessible, lower-risk populations, whereas PPRV transmission may persist in inadequately vaccinated high-risk populations despite very high overall coverage. These findings demonstrate that crude population-level coverage should not be equated with effective herd protection and support risk-based vaccination strategies that account for heterogeneous transmission patterns.

Multiple outbreaks have been documented in vaccinated herds where goat kids aged 3–6 months succumbed while adults remained protected [36]. These outbreaks demonstrate that herd immunity at the population level does not guarantee protection for the kid subpopulation [7].

Maternal antibody interference represents an additional source of heterogeneity because young animals vaccinated during the period of substantial maternal antibody persistence may fail to develop adequate vaccine-induced immunity. As maternal antibodies subsequently decline, these animals may enter a susceptible window despite having been included in vaccination campaigns. Consequently, high nominal vaccination coverage may overestimate effective population immunity if vaccine take is reduced in a substantial proportion of young animals.

For eradication by 2030, vaccination strategies must move from one-size-fits-all schedules to age-stratified, context-specific approaches that account for MA dynamics in goat kids [35,37]. Ensuring that young animals receive effective vaccine-induced protection after maternal antibody interference has sufficiently declined may help close otherwise hidden immunity gaps and improve the probability of sustained transmission interruption.

### 4.1. Modeling Herd Immunity in the Presence of MA Interference

Mathematical models can account for MA interference by incorporating age-dependent immunity profiles [7]. Age-structured models divide the population into age cohorts, each with different MA decay kinetics and vaccination response probabilities. Such models can predict the proportion of goat kids in the susceptibility window at any given time, the vaccination coverage required to achieve effective herd immunity, and the optimal vaccination age for different production systems [35,38].

Key modeling insights indicate that even 90% coverage in goat kids may produce only 50–70% effective immunity if vaccination occurs during the susceptibility window [7,39]. Delaying vaccination to 6 months reduces the required coverage threshold by 15–20%, while booster vaccination reduces the effective coverage gap by 30–40% [40]. However, models require input data on MA decay rates, protective thresholds, and field challenge intensity data that are currently sparse for many regions, including Pakistan [7,41].

### 4.2. Categorization of Quantitative Estimates

To improve transparency and enable readers to assess the strength of the evidence, quantitative estimates presented in this review are categorized according to their source: experimental data derived from controlled vaccination or challenge studies, field data derived from observational studies conducted under natural or field conditions, and model-derived estimates derived from mathematical or statistical models.

The following table summarizes selected quantitative claims, their sources, evidence type, and relevant study characteristics (Table 6).

## 5. Strategies to Overcome Maternal Antibody Interference

Several strategies can mitigate or bypass maternal antibody interference in goat kids. These range from schedule adjustments to vaccine innovations.

### 5.1. Schedule-Based Strategies

Delayed vaccination is the simplest approach. Vaccinating at 6–9 months of age, when MAs have declined to undetectable levels, ensures seroconversion in nearly all goat kids [23]. However, this leaves goat kids unprotected during the critical 2–5-month susceptibility window, which is acceptable only in low-risk environments in higher-risk endemic settings, alternative or supplementary strategies (e.g., booster schedules) may be more appropriate, though all approaches require field validation.

Booster vaccination schedules have also been proposed as a means of improving seroconversion rates. A primary dose followed by a booster at a later age can potentially increase the proportion of responders [23]. In a study of Sirohi goat kids, a single dose at 4 months resulted in 94.9% seroconversion at 28 days’ post-vaccination, though the presence of maternal antibodies negatively affected the response [23]. This highlights the potential benefit of a booster strategy, although direct comparative studies of single-dose versus booster schedules are limited. The specific ages reported in the literature (e.g., 4 months followed by 7 months, or 5–6 months followed by 8–9 months) vary across studies and should not be interpreted as universal recommendations. Rather, they illustrate the principle that booster strategies may help close the immunity gap. The optimal timing must be determined according to local maternal antibody kinetics, breed, colostrum management, and epidemiological conditions.

Targeted vaccination based on risk assessment optimizes resource use. goat Kids born from young dams (lower MAs) can be vaccinated earlier (3–4 months), while goat kids from older dams (higher MAs) require later vaccination (6–7 months). Herds with good colostrum management may need delayed vaccination compared to herds with poor colostrum intake [7]. Modeling studies provide additional insights into optimal vaccination strategies. Key model-derived insights indicate that vaccination during the susceptibility window may result in substantially lower effective immunity than suggested by coverage rates alone [7,35,39]. Delaying vaccination to later ages (e.g., 6 months) and implementing booster strategies have been shown in modeling studies to improve effective immunity, though the exact quantitative effects depend on local epidemiological and demographic parameters [7,39,40]. These are model-derived estimates and require field validation.

### 5.2. Vaccine-Based Strategies

Thermostable vaccines reduce logistical barriers but do not solve MA interference. However, improved thermos ability allows vaccination campaigns in remote areas where multiple booster visits are difficult [38].

Novel vaccine platforms under development offer theoretical potential to reduce MA interference. Recombinant vector vaccines (e.g., Capri poxvirus-vectored PPR) may offer an advantage in the presence of maternal antibodies because the vector particle itself does not display PPRV surface glycoproteins (H/F), which are the primary targets of neutralizing antibodies. However, these vectors are replication-competent and express PPRV antigens after vector replication, which may still be subject to immune recognition. Furthermore, pre-existing immunity to the vector backbone could limit vaccine efficacy. Non-replicating vaccines, which do not require viral replication, represent a different platform with distinct mechanisms; however, this remains a theoretical advantage that requires experimental confirmation, as maternal antibodies may affect vaccine responses through mechanisms beyond viral neutralization, including epitope masking and Fc receptor-mediated suppression [42]. Experimental evidence for recombinant vaccines in PPR remains limited to early-stage studies, and none are currently field-ready for PPR eradication [42]. Field validation is needed to confirm their efficacy in the presence of maternal antibodies under real-world conditions.

Intranasal vaccination in goat kids may partially overcome interference by stimulating local mucosal immunity independent of systemic Mas [24] demonstrated that intranasal administration induced protective immunity even in the presence of moderate MA titers [24]. However, this remains an experimental approach requiring further field validation.

### 5.3. Management-Based Strategies

Several management-based strategies have been proposed to address maternal antibody interference, though their applicability varies. Delayed colostrum feeding (withholding colostrum for 6–12 h after birth) reduces initial MA titers, shortening the interference period.

Historically, this has been used experimentally to reduce maternal antibody interference; however, this practice is controversial and is not recommended in field settings because it increases the risk of failure of passive transfer [26].

Improved colostrum management ensures consistent MA levels, making vaccination timing more predictable. However, it does not reduce interference; it simply reduces variability [7].

Candidate strategy recommendation: In practice, a combination of delayed primary vaccination (5–6 months) plus a booster (8–9 months) appears promising based on available evidence. However, this should be considered a candidate strategy requiring field validation, as optimal timing depends on maternal antibody kinetics, breed, colostrum management, and local transmission risk [23]. Among available strategies, a combined delayed-primary plus booster vaccination approach currently offers a practical solution under endemic field conditions (Figure 3), but its effectiveness should be validated through field studies in different epidemiological settings before being adopted as a standard recommendation.

## 6. Field and Socio-Economic Challenges

Even when optimal vaccination strategies exist, their implementation faces substantial field and socio-economic barriers.

Farmer awareness is a major constraint. Knowledge, Attitude, and Practice (KAP) studies across Africa and Asia reveal that most smallholder farmers do not understand maternal antibody interference. They vaccinate goat kids once at the same time as adults, assuming protection. Farmers rarely recognize the susceptibility window or the need for delayed or booster vaccination [43].

Vaccine access remains limited in remote areas. Cold chain infrastructure is inadequate in many pastoral and agro-pastoral systems. Live attenuated PPR vaccines require strict temperature control (2–8 °C). Thermostable formulations exist but are not widely deployed. Consequently, vaccination campaigns occur intermittently, not at biologically optimal times [44].

Cross-border animal movement undermines vaccination efforts. Goats and sheep move across national boundaries for trade, transhumance, and drought escape. Under-vaccinated or unvaccinated goat kids from neighboring regions introduce PPR into vaccinated herds, perpetuating endemicity. Synchronized regional vaccination campaigns are rare but essential [45]. Figure 4 illustrates this transboundary dynamic, with emphasis on the Pakistan–Afghanistan–Iran.

Cost constraints affect both governments and farmers. Booster schedules double the vaccine doses and delivery costs per kid. Delayed vaccination requires separate campaigns for goat kids versus adults, increasing operational expenses. Poor governments prioritize coverage over precision, opting for single annual campaigns that inevitably fail for goat kids [9].

Mixed-age herd management complicates age-based strategies. Farmers cannot easily isolate goat kids for separate vaccination without additional labor and infrastructure. Community vaccinators often lack age recording systems, making targeted kid vaccination logistically difficult [1].

Colostrum management is poor in many systems. Farmers may not understand the critical 24-h window for colostrum intake or the relationship between dam vaccination and kid protection. Goat kids are sometimes sold or separated from does early, potentially affecting colostrum intake. This can contribute to variable maternally acquired antibody (MA) levels, which may complicate the application of a fixed vaccination schedule [7].

Misaligned incentives exist. National eradication programs measure success by vaccine doses administered and crude coverage rates, not by effective immunity in the kid subpopulation. Reporting systems rarely track age-specific seroconversion. Consequently, MA interference remains invisible to policymakers. Bridging the gap between biological recommendations and field realities remains the central challenge for PPR eradication programs [38]. 

### Gender and Social Dimensions

Women are often the primary goat keepers in many smallholder systems across Africa and Asia. They are responsible for feeding, health care, and marketing of goats. However, women frequently have limited access to veterinary services, extension programs, and vaccination campaigns. Key gender-related challenges include limited access to information about optimal vaccination timing and colostrum management, vaccination campaigns often scheduled at times that conflict with women’s domestic responsibilities, reduced decision-making power regarding vaccination expenditure, and extension materials that are not accessible to women with low literacy [46].

To address these challenges, several recommendations are proposed. Gender-sensitive extension programs specifically targeting women goat keepers should be designed and implemented. KAP studies should be disaggregated by gender to better understand the specific knowledge gaps and barriers faced by women. Simple, visual education materials such as pictorial guides should be developed for low-literacy audiences. Additionally, vaccination campaigns should be scheduled at times convenient for women to ensure their participation and engagement [43].

## 7. Research Gaps and Future Directions

Addressing maternal antibody interference requires targeted research across epidemiological, immunological, and operational domains. Despite decades of PPR research, critical knowledge gaps remain regarding maternal immunity in goat kids. Addressing these is essential for eradication by 2030 [9].

### 7.1. Epidemiological Research Gaps

Limited longitudinal evidence on maternal antibody persistence in Pakistan represents an important research gap. Most available evidence reviewed in this manuscript originates from other regions, including South Asia and Africa. Pakistan has diverse goat breeds and production systems, but maternal antibody kinetics and vaccine responsiveness under field conditions remain insufficiently characterized. A recent systematic review and meta-analysis by [47] reported an overall pooled PPR prevalence of 51% (95% CI: 42–60) in small ruminants across Pakistan, with regional variation ranging from 44% in Khyber Pakhtunkhwa to 61% in Sindh. Serological surveys have provided indirect evidence of maternal antibody dynamics; for instance, studies from Azad Jammu and Kashmir and Sindh have reported lower seropositivity in young kids compared to adults, consistent with the expected decline of maternally derived antibodies [48]. However, no published study has systematically tracked maternal antibody decay in Pakistani goat kids under field conditions across different breeds, production systems, and ecological zones.

Pakistan is therefore highlighted as a priority for future research rather than as a region with established Pakistan-specific maternal antibody evidence. Longitudinal studies across representative breeds and production systems are needed to characterize maternal antibody decay, identify locally relevant susceptibility periods, and evaluate the feasibility of age-stratified vaccination strategies. Understanding breed-specific MA kinetics in Pakistani goat breeds (e.g., Beetal, Teddy, Kamori, Dera Din Panah) would help determine whether vaccination schedules developed elsewhere are appropriate for local conditions. Region-specific vaccination timing is unknown for most production systems. The optimal age for primary vaccination varies with breed, nutrition, colostrum management, and endemicity. Generic recommendations (e.g., “vaccinate at 4–6 months”) are insufficient. Research should determine location-specific susceptibility windows and validate delayed booster schedules [6].

Cross-border harmonization research is minimal. Studies on MA dynamics in transboundary animal movement corridors (e.g., Pakistan-Afghanistan, Pakistan-Iran borders) are absent. Understanding how mixing of herds with different vaccination histories affects kid immunity would inform regional eradication strategies [25].

### 7.2. Immunological Research Gaps

The interference threshold remains undefined for PPR in goat kids. No identified study directly establishes the antibody titre above which administration of live attenuated PPR vaccine fails or is substantially impaired in inducing an active immune response. This represents one of the most consequential unresolved questions for optimizing vaccination schedules in young animals.

Future studies should prospectively measure pre-vaccination maternal antibody levels together with post-vaccination vaccine take and seroconversion to determine the antibody level above which vaccination fails or is substantially impaired in inducing an active immune response. Such studies should use standardized serological methods and clearly distinguish antibody persistence from vaccine-induced active immunity.

Diagnostic cut-offs need standardization. No universally accepted protective antibody titer exists for goat kids against PPR [6,15]. Research should determine the minimum neutralizing antibody level that correlates with protection from clinical disease and mortality, stratified by circulating virus lineage.

Colostrum quality studies are lacking. The relationship between colostral antibody concentration, volume ingested, timing of intake, and subsequent MA persistence in goat kids is poorly quantified for PPR. Research should establish colostrum feeding guidelines specifically for PPR control [26].

Duration of protective immunity after vaccination in the presence of MAs is not well characterized. Studies should determine how long vaccinated goat kids are protected if they seroconverted despite low-level MA interference [16].

### 7.3. Operational Research Gaps

Candidate serological approaches to distinguish maternal from vaccine-induced antibodies—such as IgM-specific ELISA, IgG avidity indices, and paired sera testing—exist and are used in other viral systems; however, their validation and field application for PPR in goat kids remain a priority. Research should focus on validating IgM-specific ELISA and IgG avidity indices for PPR serology, establishing field protocols for paired sera testing to assess longitudinal antibody kinetics, and developing DIVA-capable vaccine platforms as a complementary approach for distinguishing infection from vaccination.

Rapid pen-side tests are urgently needed. Farmers and vaccinators cannot currently measure MA status before vaccinating. A rapid, affordable test (similar to lateral flow devices for rinderpest) would enable individualized vaccination timing. Development and field validation of such tests should be a research priority [28].

Modeling and surveillance are underexplored. Mathematical models can predict MA decay curves based on initial titers, half-life estimates, and field challenge intensity. Such models could generate age-specific vaccination calendars for different herd types. However, models require input data that are currently sparse [39].

Vaccine platform innovation continues to be needed. While recombinant and non-replicating vaccines bypass MA interference, none are commercially available for PPR. Field trials comparing existing live vaccines with next-generation candidates in kid populations with varying MA levels are required [49].

## 8. Conclusions

Maternal antibody interference is a critical but often overlooked barrier to PPR eradication in goat kids. This review has demonstrated that the level and duration of MAs are highly variable, influenced by vaccine-related factors (strain, dose, route, frequency), host-related factors (colostrum quality, nutrition, age of dam, breed, sex), viral and environmental factors (circulating strains, management systems), and diagnostic limitations. The window of susceptibility typically between 2 and 5 months of age leaves goat kids vulnerable to infection while still interfering with live vaccination. Consequently, fixed-age vaccination campaigns produce immunity gaps, undermining herd protection in the most susceptible age cohort.

Several key conclusions emerge from this review. A one size-fits-all vaccination schedule is inadequate for PPR eradication, and context-specific strategies including delayed vaccination, booster schedules, and targeted approaches based on risk factors—are essential. Field realities such as poor colostrum management, limited vaccine access, cross-border animal movement, and low farmer awareness severely constrain implementation. Furthermore, diagnostic tools for field measurement of MAs are currently unavailable, forcing blind vaccination based on fixed-age schedules that inevitably fail for a proportion of goat kids.

Based on these conclusions, several policy-oriented recommendations are proposed. National PPR eradication programs should adopt age-stratified vaccination protocols rather than single-age campaigns. Booster vaccination strategies should be considered for evaluation in endemic settings, with timing informed by local maternal antibody kinetics, breed, colostrum management, and transmission risk. Given the additional logistical and economic requirements associated with booster campaigns, their effectiveness, feasibility, and cost-effectiveness should be evaluated under field conditions before consideration for routine implementation. Farmer education on colostrum management and the susceptibility window must accompany vaccination campaigns to ensure proper implementation. Finally, cross-border harmonization of vaccination schedules is necessary to prevent reintroduction of the virus through transboundary animal movement.

### A Call for a Paradigm Shift

Achieving PPR eradication by 2030 requires shifting focus from crude coverage to effective immunity in young animals. Research priorities should include longitudinal MA studies in underrepresented regions (including Pakistan), development of rapid pen-side diagnostic tests, standardization of protective antibody cut-offs, and field validation of novel vaccine platforms that bypass MA interference. Without addressing maternal immunity as a critical barrier, global PPR eradication will remain elusive. The time for a paradigm shift from counting doses administered to measuring effective immunity achieved is now.

## Figures and Tables

**Figure 1:**
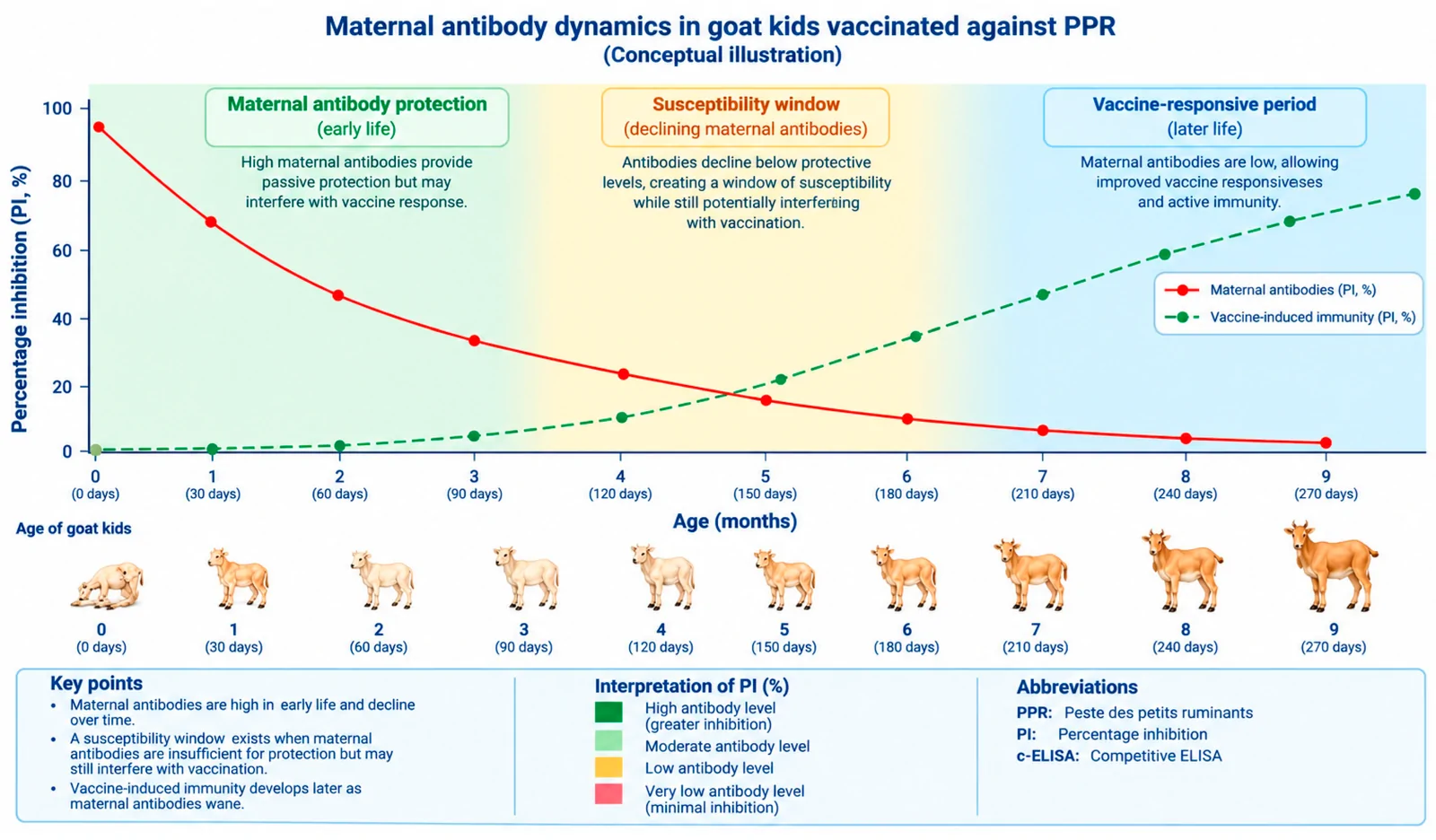
Conceptual representation of maternal antibody dynamics in goat kids vaccinated against PPR. The x-axis represents age (0–9 months), and the y-axis represents percentage inhibition (PI, %) measured by competitive ELISA. The figure illustrates three conceptual phases: a maternal antibody protection phase (early life), during which maternal antibodies provide passive protection but may interfere with vaccine response; a susceptibility window (declining maternal antibodies), during which antibodies decline below protective levels yet may still interfere with vaccination; and a vaccine-responsive period (later life), during which maternal antibodies are low, allowing improved vaccine responsiveness and active immunity. The transitions between phases are gradual and variable and should not be interpreted as fixed age or serological cut-offs. The figure is an original conceptual illustration prepared by the authors based on patterns reported in published studies, including [11,12].

**Figure 2:**
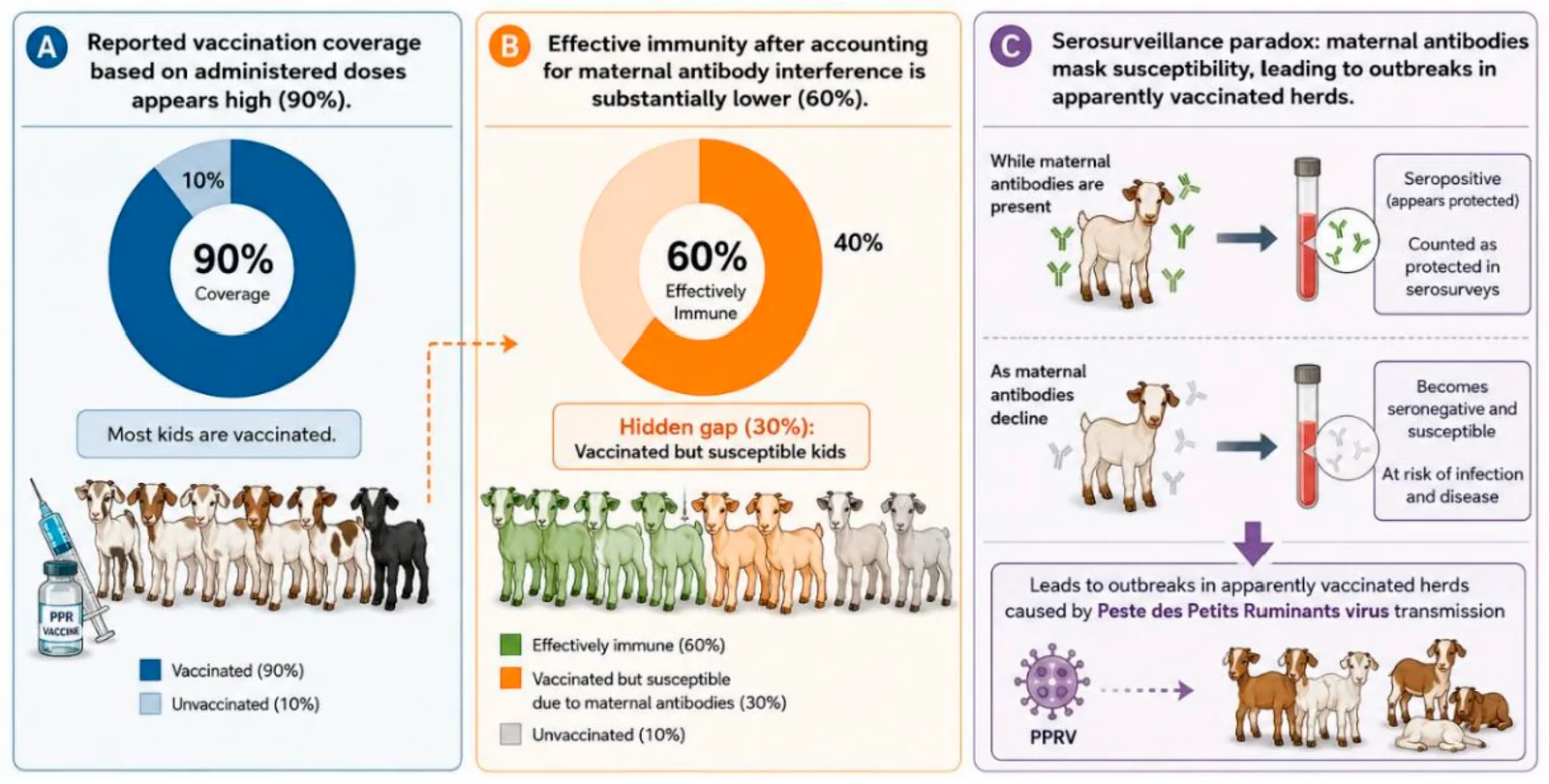
Conceptual illustration of the hidden immunity gap and serosurveillance paradox caused by maternal antibody interference in goat kids. (**A**) Reported vaccination coverage based on administered doses is illustrated as (90%) for conceptual purposes. (**B**) Effective immunity after accounting for maternal antibody interference is illustrated as (60%), revealing a hidden gap (30%) of vaccinated but susceptible goat kids. (**C**) Goat kids with maternal antibodies test seropositive and are counted as protected; however, once maternal antibodies decline, they become susceptible, contributing to outbreaks in apparently vaccinated herds. The numerical values shown are illustrative and hypothetical; they do not represent actual epidemiological estimates. Real-world gaps will vary with local conditions, vaccination programs, and epidemiological settings.

**Figure 3:**
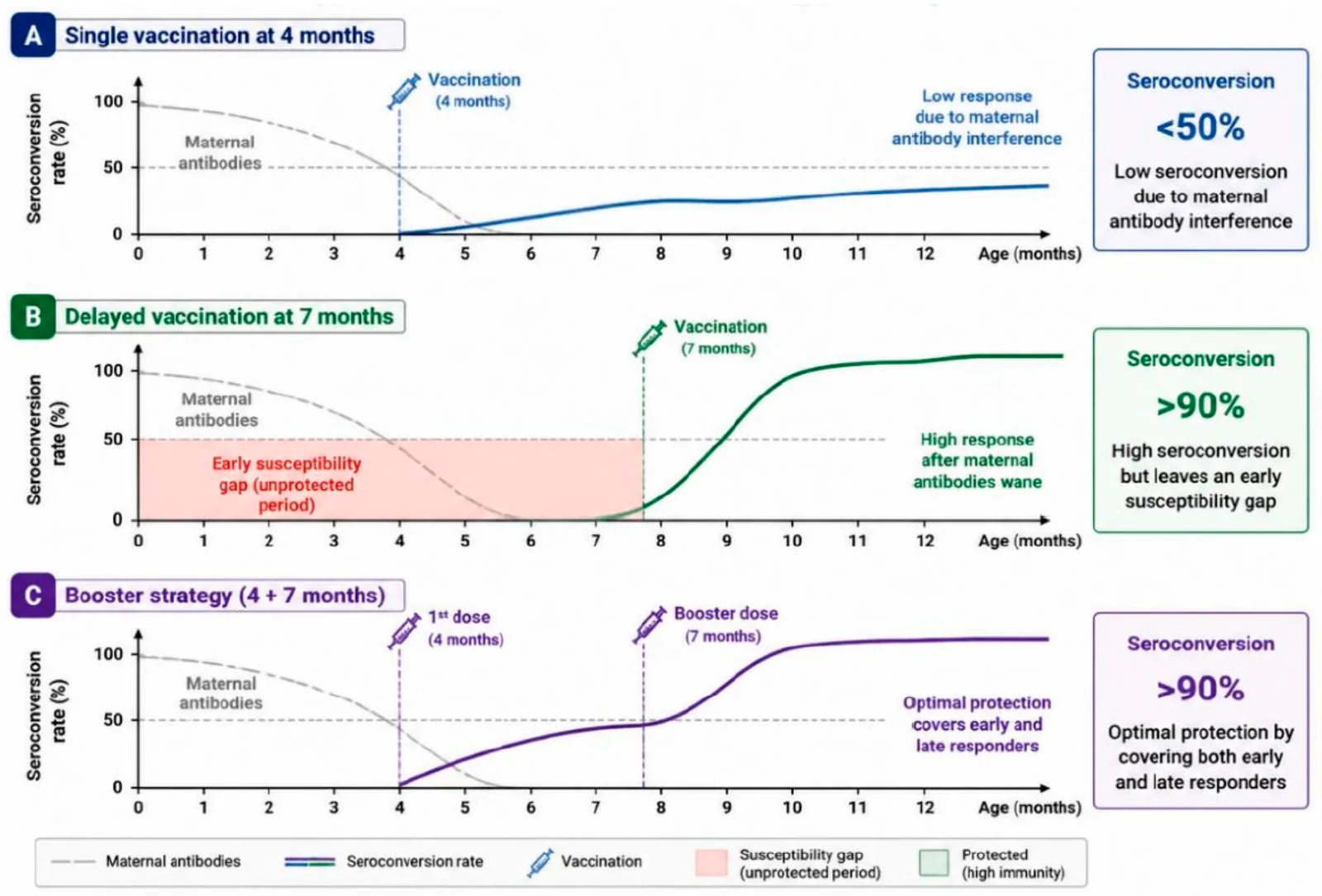
Conceptual comparison of vaccination strategies in goat kids. (**A**) Single vaccination at 4 months results in low seroconversion (<50%) due to maternal antibody interference. (**B**) Delayed vaccination at 7 months results in high seroconversion (>90%) but leaves an early susceptibility gap. (**C**) Booster strategy (4 + 7 months) achieves optimal protection (>90%) by covering both early and late responders. The seroconversion values shown are illustrative and based on general trends observed in the literature; they are not derived from a single empirical study. Actual seroconversion rates will vary with vaccine strain, dose, route, breed, and epidemiological context.

**Figure 4:**
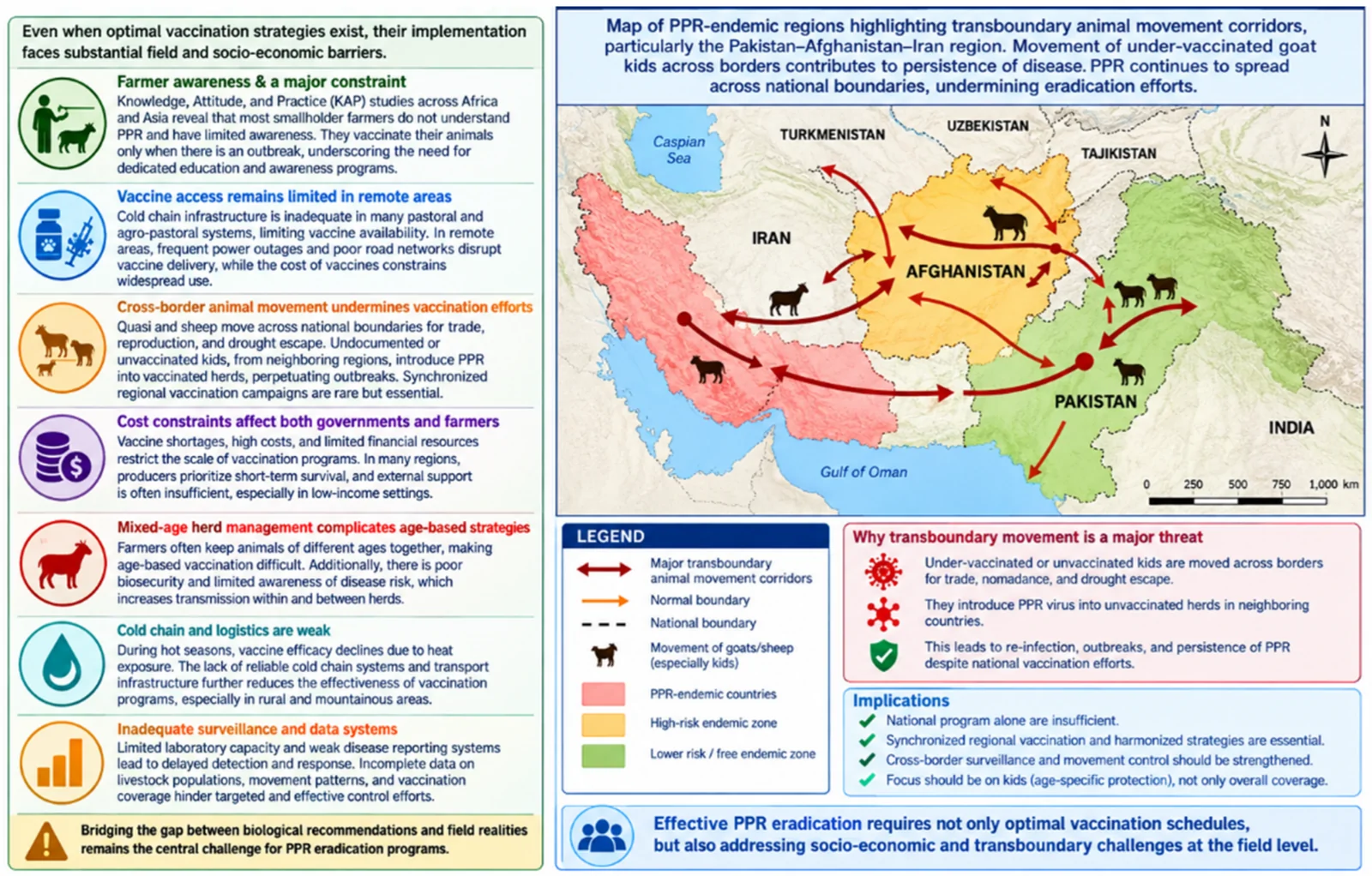
Original map of PPR-endemic regions highlighting transboundary animal movement corridors, with emphasis on the Pakistan–Afghanistan–Iran region. The map was created by the authors based on FAO/WOAH PPR distribution data and documented livestock trade and transhumance routes in South Asia and the Middle East. The highlighted corridors represent known transboundary animal movement routes used for livestock trade and seasonal migration. The Pakistan–Afghanistan–Iran corridor is highlighted because of its importance to the authors’ stated research gap and the need for region-specific studies in this area. However, other major transboundary corridors (e.g., West Africa, East Africa, the Horn of Africa, and South Asia) are equally important for regional PPR persistence and should also be considered in eradication strategies. This map is a conceptual illustration and does not represent comprehensive movement data for all regions.

**Table 2:** Host-related factors potentially affecting maternal antibody persistence in goat kids.

Factor	Comparison/ Category	Effect on MA Level	Potential Effect on Persistence	Evidence/Interpretation	Supporting Reference(s)
Colostrum intake	Adequate vs. poor	Higher vs. lower	May influence duration	Major determinant of passive antibody transfer	[7,23]
Age of dam	Younger vs. older	Potential variation	Potential variation	Limited evidence; should not be interpreted as an established PPR-specific effect	[10]
Sex	Female vs. male	Potential variation	Potential variation	Evidence for a specific effect on PPR maternal antibody persistence is limited	[10]
Colostrum timing	Early vs. delayed intake	Earlier intake generally increases passive antibody acquisition	May influence persistence by affecting initial antibody levels	Timing of colostrum intake is important for passive antibody transfer; PPR-specific evidence remains limited	[26,27]

**Table 3:** Diagnostic tools for maternal antibody detection.

Parameter	c-ELISA	VNT (Virus Neutralization Test)
Principle	Detects total antibodies (binding)	Detects neutralizing antibodies
Specificity	Moderate (88.24% in goats)	High (gold standard)
Time Required	2–4 h	3–5 days
Cost	Low	High
Technical Requirement	Basic lab	Advanced lab + cell culture
Field Applicability	High	Very low
Distinguish MA vs. Vaccine Antibodies	No	No (functional but not origin-specific)
Use in Surveillance	Widely used	Limited
Suitability for Vaccination Timing	Limited	Better but impractical
Supporting Reference(s)	[6,27]	[27,28]

**Table 4:** Strategies to overcome maternal antibody interference. Feasibility ratings reflect practical implementation in large-scale vaccination programs; individual farm-level practices (e.g., colostrum management) may differ in their practicality and risk profiles.

Strategy	Mechanism	Effectiveness	Feasibility	Limitations	Supporting References
Delayed Vaccination (6–9 months)	Avoids MA interference	High	Moderate	Leaves early-life gap	[6]
Booster Vaccination (4 + 7 months)	Covers both early and late responders	Very high (>90%)	Moderate	Higher cost/logistics	[23]
Targeted Vaccination	Based on MA risk factors	High	Low–moderate	Requires data/monitoring	[7]
Intranasal Vaccination	Induces mucosal immunity	Moderate–high	Experimental	Limited field validation	[25]
Thermostable Vaccines	Improves delivery	Indirect benefit	High	Does not solve MA interference	[33]
Novel Vaccines (Recombinant)	Bypass MA neutralization	Promising	Low (not field-ready)	Still under development	[34]
Colostrum Management	Controls initial MA level	Variable	Low	Risk to neonates	[7]

**Table 5:** Categorization of factors by strength and directness of evidence.

Factor	Evidence Level	Supporting References	Justification
Colostrum quality and timing	Moderate	[15,23]	Multiple studies show colostrum is a key determinant of MA titers, but direct causal evidence is limited
Vaccine strain	Moderate	[23,24]	Some strain-specific differences reported, but limited direct comparisons
Vaccine dose	Moderate	[6]	Higher doses produce stronger MA responses, but limited dose–response studies
Route of administration	Moderate	[24]	Subcutaneous vs. intranasal compared; limited intramuscular data
Dam vaccination timing	Moderate	[6,7]	4–6 weeks pre-partum appears optimal; limited comparative studies
Breed	Weak/Limited	[15,23]	Some breed differences reported, but limited systematic comparisons
Sex	Weak/Limited	Limited evidence	Few studies have examined sex differences in MA persistence
Age of dam	Weak/Limited	Limited evidence	Few studies have systematically compared MA transfer by dam age
Nutrition	Speculative	No direct PPR-specific studies	Plausible based on general immunology; no PPR-specific evidence
Environmental factors	Speculative	No direct PPR-specific studies	Plausible but lacking direct evidence
Management systems Interference threshold	Speculative No direct evidence identified	No direct PPR-specific studies No identified study directly defines the antibody threshold for vaccine interference in PPR goat kids	Plausible but lacking direct comparative studies The antibody level above which residual maternal antibodies impair live PPR vaccine take has not been experimentally established

**Table 6:** Categorization of selected quantitative estimates by evidence type and study characteristics.

Quantitative Claim	Estimate	Evidence Type	Source	Study Characteristics
Sirohi goat kids with protective titres before vaccination	3.29%	Experimental	[23]	Sirohi breed, India; *n* = 374; Sungri/96 vaccine; organized farm
Maternal antibody titres became negative	By 100 days	Experimental	[7]	Saanen breed, Iran; *n* = 20; Nigeria 75/1 vaccine
Average maternal antibody titres became negative	70–100 days	Experimental	[7]	Saanen breed, Iran; *n* = 20; Nigeria 75/1 vaccine
Maternal antibodies fell below the protective threshold in goat kids	4.5 months	Field	[15]	Kirdi breed, Cameroon; on-station observational study
Maternal antibodies remained detectable but were below the protective level	Up to 6 months; below protective level by the fourth month	Experimental	[6]	Goats, India; lineage IV vaccine

## Data Availability

No new datasets were generated or analyzed in this review. All data presented are derived from previously published studies and are cited accordingly.

## Abbreviations

The following abbreviations are used in this manuscript:

c-ELISA	Competitive enzyme-linked immunosorbent assay
FAO	Food and Agriculture Organization
IgG	Immunoglobulin G
KAP	Knowledge, Attitude, and Practice
MA	Maternal antibody
PI	Percentage inhibition
PPR	Peste des petits ruminants
PPRV	Peste des petits ruminants virus
SC	Subcutaneous
TCID_50_	50% tissue culture infective dose
VNT	Virus neutralization test
WOAH	World Organisation for Animal Health
